# Cross-Modal Recruitment of Primary Visual Cortex by Auditory Stimuli in the Nonhuman Primate Brain: A Molecular Mapping Study

**DOI:** 10.1155/2012/197264

**Published:** 2012-06-21

**Authors:** Priscilla Hirst, Pasha Javadi Khomami, Amol Gharat, Shahin Zangenehpour

**Affiliations:** ^1^Department of Psychology, McGill University, Montreal, QC, Canada H3A 1B1; ^2^School of Optometry, Université de Montréal, Montreal, QC, Canada H3T 1P1

## Abstract

Recent studies suggest that exposure to only one component of audiovisual events can lead to cross-modal cortical activation. However, it is not certain whether such crossmodal recruitment can occur in the absence of explicit conditioning, semantic factors, or long-term associations. A recent study demonstrated that crossmodal cortical recruitment can occur even after a brief exposure to bimodal stimuli without semantic association. In addition, the authors showed that the primary visual cortex is under such crossmodal influence. In the present study, we used molecular activity mapping of the immediate early gene zif268. We found that animals, which had previously been exposed to a combination of auditory and visual stimuli, showed increased number of active neurons in the primary visual cortex when presented with sounds alone. As previously implied, this crossmodal activation appears to be the result of implicit associations of the two stimuli, likely driven by their spatiotemporal characteristics; it was observed after a relatively short period of exposure (~45 min) and lasted for a relatively long period after the initial exposure (~1 day). These results suggest that the previously reported findings may be directly rooted in the increased activity of the neurons occupying the primary visual cortex.

## 1. Introduction

Sensory processing of environmental stimuli starts at the level of specialized peripheral organs (e.g., skin, eyes, ears, etc.) and follows segregated information processing pathways in the central nervous system. To have coherent and unified percepts of multimodal events (e.g., containing sound and light as in the case of a moving car or a vocalizing conspecific), sensory information across these apparently divergent pathways needs to be integrated. Integration of information across two or more sensory channels involves multiple subcortical structures [1–4], as well as cortical regions (e.g., parietal cortex [5, 6], the superior temporal sulcus [7–9], and the insular cortex [10, 11]).

In more recent accounts of sensory processing, interactions between modality-specific channels are emphasized. For example, recent work shows that the activity of unisensory cortices can be under a cross-modal influence. For example, recent studies show that a primary sensory cortex can be under inhibitory [12–17] or excitatory cross modal influence [18–21]. Findings from single-unit recordings of visual influence on early auditory cortical processing [22, 23] also demonstrate that activity in nominally unisensory auditory cortex can be modulated by the presence of a concurrent visual stimulus; similar data have been reported in human neuroimaging studies, where modulation of one sensory cortex occurs due to multisensory costimulation (for review see [24–26]).

In addition, it has been shown through several lines of evidence that a stimulus presented through only one sensory modality affects the processing and perception of a stimulus presented in another modality. The flash-beep illusion introduced by Shams et al. [27] is a clear example of such multisensory interactions whereby the *perceived* number of visual flashes appears to be positively linked to the *actual* number of simultaneous beeps for a single flash of light. Subsequent work on the neurophysiological underpinnings of this illusion has revealed the involvement of the primary visual cortex (V1) and/or other early visual cortical areas [28–31]. These phenomena occur over very short time scales (typically a few tens/hundreds of milliseconds) and most likely reflect a process of integration of the information coming from the two modalities.

When the time scale of such interactions is expanded beyond minutes and hours, one comes across situations where a stimulus presented in only one sensory modality recruits regions pertaining to a different modality (such as in the case of lipreading [32–35] or the case where visual cortical areas have been shown to respond to auditory components of typically bimodal events with a close semantic relationship, such as tools and their sounds [7, 8] or voices and faces [36, 37]). In addition, a number of neuroimaging studies have also directly investigated the nature of cross-modal activity following learning or conditioning paradigms in which arbitrary pairings of unrelated auditory or visual stimuli [21, 38, 39] are shown to lead to cross-modal recruitment. 

Immediate early genes (IEGs) are a group of genes that are transiently activated following sensory stimulation [40]. IEG zif268 encodes a transcription factor that has a regulatory role in neuronal processes such as excitability, neurotransmitter release, and metabolism [40], and the time course of its mRNA and protein expression has been studied [41]. The inducible expression of IEG protein products is commonly used to map neural activity at the cellular level by immunohistochemistry (for extensive reviews see [40, 42–47]). One benefit of using zif268 expression in activity mapping is that is has considerable staining reliability and availability of antibodies [48]. Furthermore, cellular resolution mapping provides a precise localization of neural activity over large areas of the cortex. This provides a framework for large-scale analysis with single-cell resolution, a combination that is difficult to achieve by any other method [48, 49]. Since analysis is performed postmortem, the technique permits the experimental animals to be treated with a flexible behavioral schedule. Unlike functional neuroimaging or electrophysiological techniques, a molecular mapping study requires little preparatory procedures. The animals are therefore permitted to behave more naturally in an unrestricted environment prior to experimentation [48, 49]. 

Molecular mapping can be used to reveal activation maps of brain areas in response to each component of a stimulus sequence. This allows for the visualization of segregated populations of neurons that are each responsive to a different part of the sequence [41]. Given that the time course of zif268 induction has been determined, if an animal is exposed to a compound stimulus followed by euthanasia at a precise time point, one can establish the point within the sequence that a given population of neurons became active by assessing the level of zif268 expression in the tissue. Based on the temporal properties of zif268 induction, neurons that respond to the first part of the compound stimulus should contain detectable levels of the protein, whereas neurons responsive to the second part of the sequence should not [41]. On this basis, if an animal is preexposed to the bimodal stimulus and subsequently experiences auditory followed by visual stimulation before euthanasia, zif268 expression in the primary visual cortex in addition to the auditory cortex would constitute evidence for cross-modal recruitment.

A recent functional positron emission tomography (PET) study [50] has explored the circumstances under which cross-modal cortical recruitment can occur with unimodal stimuli in the absence of a semantic association, an explicit conditioning paradigm, or prolonged, habitual cooccurrence of bimodal stimuli. It was found that human subjects who had been preexposed to audiovisual stimuli showed increased cerebral blood flow in the primary visual cortex in response to the auditory component of the bimodal stimulus alone, whereas naïve subjects showed only modality-specific activation. The results indicate that inputs to the auditory system can drive activity in the primary visual cortex (V1) 1 day after brief exposure and persist for 16–24 hours [50]. However, due to the inherent limitations of correlating changes in blood flow to underlying neural activity as in the case of functional PET imaging, it still remains unclear whether or not the observed cross-modal phenomenon is directly linked to increased neural activity in area V1.

Thus, the goal of the present study was to investigate the previously described cross-modal recruitment of V1 in response to auditory stimuli using a more direct method of visualizing brain activity, namely, molecular activity mapping of the IEG zif268, in order to describe a more direct link between nonrelevant sensory input and cross-modal cortical activation.

## 2. Materials and Methods

### 2.1. Animals

The subjects were four adult vervet monkeys (*Chlorocebus sabaeus*). Animal experimentation, conducted in accordance with the Animal Use Protocol of the McGill University Animal Care Committee, was performed at the Behavioural Sciences Foundation on the Island of St Kitts. This facility is fully accredited by the Canadian Council on Animal Care. Externalized brain tissue was transported back to McGill University where histological processing and data analysis were conducted.

### 2.2. Apparatus and Stimuli

Both auditory and visual stimuli were presented using a Sony 37′′ LCD Digital TV with integrated stereospeakers connected to a MacBook Air computer (Apple Inc.). Monkeys were seated in the primate chair facing the center of the monitor at a viewing distance of 60 cm. 

**Figure 1:**
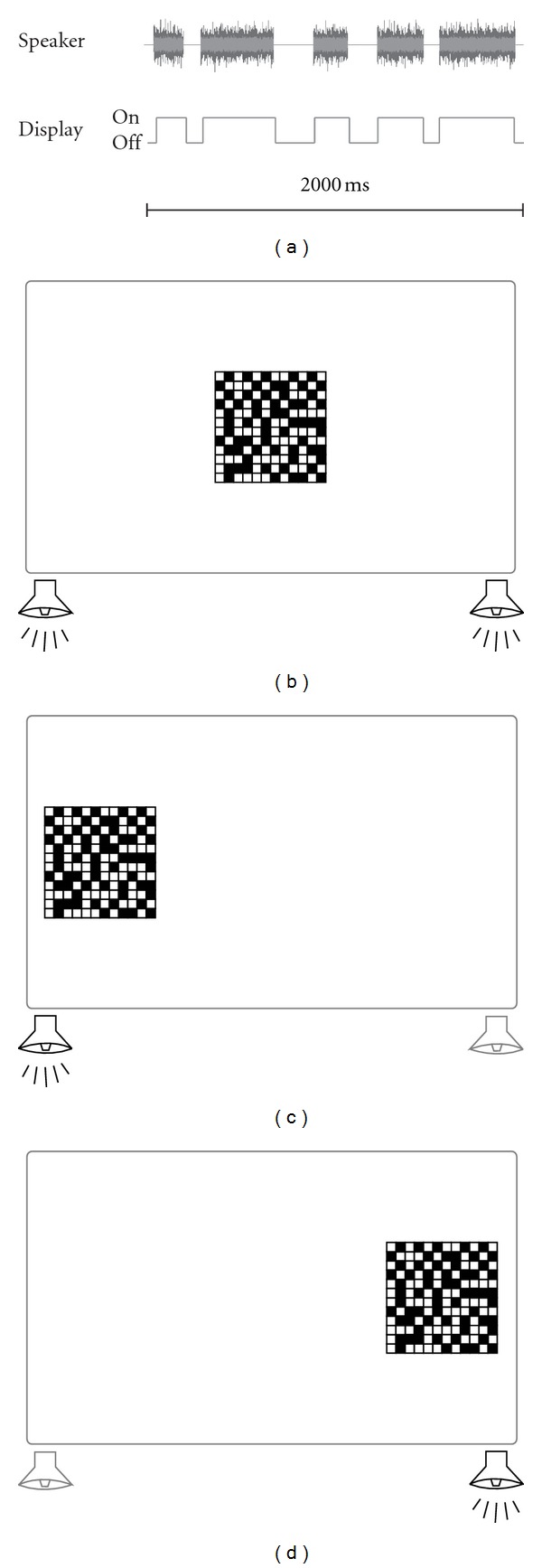
Schematics of stimuli and experimental apparatus. The stimuli presented were white-noise bursts and/or flashes of a single light source that lasted a total of 2000 msec (a), which were presented from the monitor and/or speakers at the centre (b), left (c), or right (d) side of the animals sensory space.

Auditory (A) and visual (V) stimuli (Figure 1(a)) were each presented in the form of a sequence of five elements for a total of 2 s. However, the duration of the elements was determined randomly using Matlab software on each trial such that the total length of each trial did not exceed 2 s. Three seconds of silence followed each 2 s trial. The content of the auditory stimuli was white-noise bursts whereas the visual stimuli were made up of flashes of a random dot pattern. Each 2 s stimulus was presented from one of three discrete locations (left, center, or right; Figures 1(b)–1(d)) within the confines of the sensory space as defined by the limits of the monitor and location of speakers. Those auditory and visual stimuli were also paired together and presented to a subset of monkeys as a compound bimodal stimulus for 45 minutes in order to establish implicit associations between auditory and visual modalities based on the temporal and spatial attributes of the stimuli. This category of stimuli was used to visualize the cross-modal recruitment phenomenon previously reported in humans.

### 2.3. Stimulation Procedure

Each monkey was exposed to a sequence of A followed by V stimuli (or vice versa) in order to visualize zif268 expression in response to those stimuli in primary sensory areas of the brain. The total duration of the stimulus blocks was designed such that the first stimulus lasted 60 minutes followed by 30 minutes of exposure to stimuli of the other modality. The rationale behind the choice of those periods was based on the peak of zif268 protein (henceforth Zif268) expression, which occurs at 90 minutes following the onset of sensory stimulation. Thus, two animals received the auditory followed by visual stimulation sequence (i.e., AV), and the other two animals received the reverse sequence (i.e., VA). In addition, animals in each stimulation group were further divided into two categories of Naïve (N) and Experienced (E), where N signifies the lack of experience of compound bimodal stimuli and E signifies the presence of such experience. Monkeys in group E received those compound stimuli 24 hours prior to receiving the AV or VA stimulation sequence and immediately before being euthanized for the purpose of visualizing Zif268 expression.

### 2.4. Animal Treatment and Tissue Collection

For 7 days prior to the start of this study, each day the animals experienced a two-hour habituation to the primate chair and the testing room that were subsequently used during the experiment. During these sessions they received orange- and lime-flavored juice as a reward. On the day of the experiment the monkeys were placed in the primate chair. Each animal was dark adapted while wearing a pair of foam earplugs for three hours to ensure baseline levels of Zif268 expression. Following stimulus presentation, animals received ketamine hydrochloride (10 mg/kg) sedation and were subsequently euthanized by an overdose of intravenously administered sodium pentobarbital (25 mg/kg), followed by transcardial perfusion of 0.1 M PBS to produce exsanguination. The brains were then externalized and sectioned along the coronal plane. Each hemisphere was blocked, and each block was flash-frozen in an isopentane bath cooled in a dry ice chamber and maintained at −80°C. Twenty-micrometer-thick sections were cut from the frozen blocks at −20°C on a Leica CM3050 cryostat and mounted onto Vectabond (Vector Labs) subbed glass slides. The slide-mounted tissue sections were stored at −80°C until histological processing.

### 2.5. Immunohistochemistry

Slide-mounted twenty-micron fresh-frozen tissue sections were thawed on a slide warmer at 37°C for ~5 mins. A PAP pen was used to create a hydrophobic barrier surrounding the slide-mounted tissue in order to keep staining reagents localized on the tissue section. Sections were then fixed with a 4% paraformaldehyde-in-phosphate-buffered saline (PBS) solution for 10 minutes, followed by a 5-minute PBS rinse. Sections were then washed with 0.3% hydrogen peroxide in PBS for 15 minutes to block endogenous peroxidase activity. Following another 5-minute PBS rinse, sections were acetylated in 0.25% acetic anhydride in 10 mM triethanolamine (TEA) for 10 minutes each time. Sections were then rinsed in PBS for 5 minutes and then blocked for 30 minutes with a solution of PBS and 3% normal goat serum (NGS). Each section was incubated overnight at 4°C 1 mL of rabbit anti-Zif268 polyclonal antibody (courtesy of Bravo) solution at a concentration of 1 : 10,000. Next day, sections underwent three 10-minute PBS washes, followed by incubation in a secondary antibody solution (biotinylated goat-anti-rabbit antibody diluted 1 : 500 in PBS containing 3% NGS) for 1.5 hours. The sections were then given three consecutive 10-minute PBS washes before undergoing 1-hour incubation in avidin-biotin-conjugated horseradish peroxidase (Vectastain ABC kit) solution (1 : 500). Following three subsequent 10-minute washes in PBS, the sections were treated with a 3,3-diaminobenzidine (DAB) substrate kit. The sections were then rinsed in PBS three times for 5 minutes each and subsequently underwent dehydration in graded ethanol steps, cleared in xylene, and coverslipped with Permount.

### 2.6. Cresyl Violet Stain

Cresyl violet staining of Nissl bodies was conducted on adjacent tissue sections to those that were immunostained. The purpose of the Nissl stain was to reveal anatomical landmarks for delineating auditory and visual cortical areas and to provide an estimate of the density of neurons in each brain area. The staining protocol was as follows. After removing the designated sections from freezer storage, they were left at room temperature to dry for 5 minutes. The sections then underwent graded ethanol dehydration followed by staining and rehydration. The slides were then coverslipped with Permount mounting medium and left to dry at room temperature under the fume hood.

### 2.7. Digitization of Histological Data

Following histological processing, all sections from the primary auditory and primary visual cortices were scanned using a MiraxDesk slide scanner and Mirax Viewer software (Carl Zeiss MicroImaging Inc, Thornwood, New York). Three frames were taken from each of the five scanned sections per brain area, with a sufficient number of high-magnification captures per frame (equivalent to the magnification using a 40x objective lens) to span all cortical layers. The necessary number of captures per frame depended on cortical thickness but ranged from three to six captures. Captures of the Nissl-stained scanned sections were taken from approximately the same segments of the brain areas of interest as on the corresponding immunostained scanned sections. A stereotaxic atlas was used as a reference for determining the boundaries of the areas of interest. 

### 2.8. Cell Counting and Statistical Analyses

**Figure 2:**
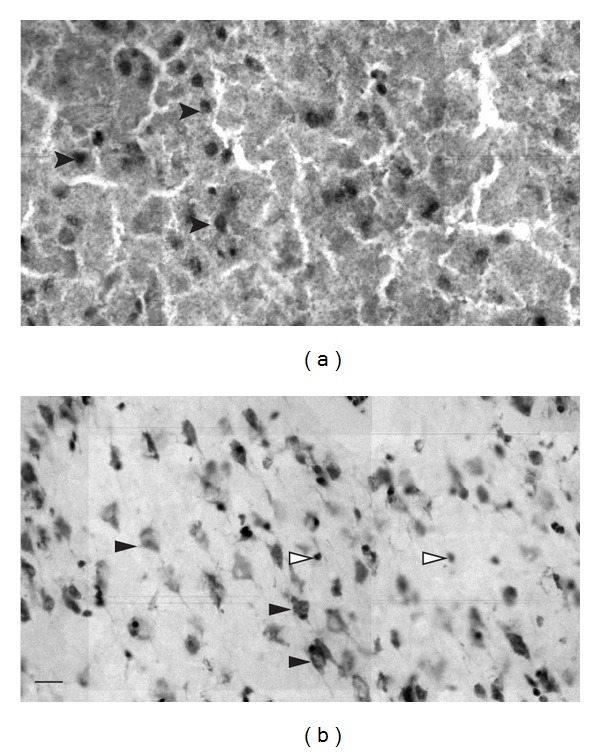
A representative sample of digitalized immunostained (a) and cresyl violet-stained (b) tissues. The black arrowheads show objects that were included in the count while the white arrowheads show objects that do not fit counting criteria and were discarded. The scale bar in panel (b) represents 20 *μ*m.

A counting frame with both inclusion and exclusion boundaries was fixed onto each captured frame. The counting frame area was approximately 310,000 pixels^2^ which converts to 38,990 *μ*m^2^ and spanned the entire length of the scanned area. Manual counts of objects were performed in each counting frame, once for the immunostained nuclei and once for the Nissl-stained cell bodies in immediately adjacent sections.  Figure 2 represents examples of counted objects. Criteria for Nissl cell counting were large endoplasmic staining with a visible nuclear envelope and one or more nucleoli. Objects such as glial cells that did not fit these criteria were discarded from the count. The density of cells was calculated by dividing the cell count by the area for each counting frame. The ratio of immunopositive neurons to the average number of neurons in the tissue section was calculated by dividing the density of immunopositive neurons by the density of Nissl-stained neurons in adjacent sections. A nonsignificant difference was found between the total cell densities of a given brain area across the four subjects. Counting was done for 40 frames from each brain area of each animal, for a total of 320 counted frames. The dependent variable was expressed as the resulting ratios and was transferred to ezANOVA statistical analysis software (http://bit.ly/vZfGCO) for the analysis of variance (ANOVA).

## 3. Results and Discussion

**Figure 3:**
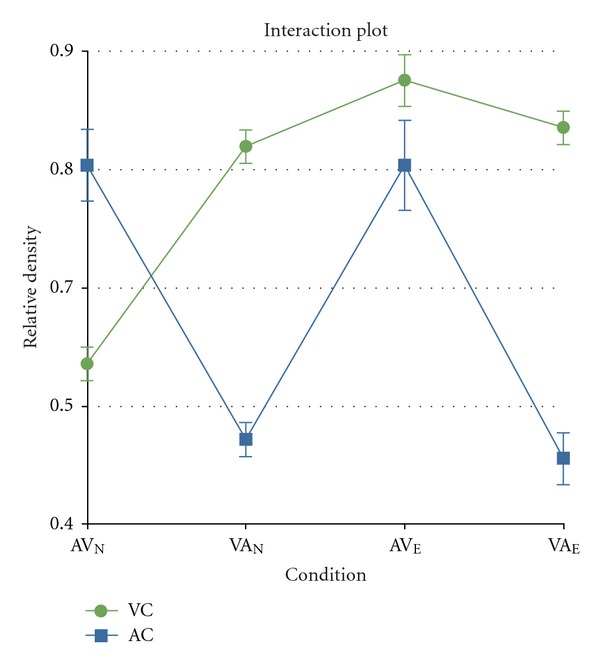
Interaction plot of the ANOVA conducted on cell counts. The primary visual cortex (V1) shows high expression of Zif268 when exposed to conditions VA_N_, AV_E_, and VA_E_ compared to baseline expression when exposed to condition VA_N_. In contrast, the primary auditory cortex (A1) shows high protein expression only when exposed to conditions AV_N_ and AV_E_ and baseline expression when exposed to VA_N_ and VA_E_. VA: visual stimulation followed by auditory stimulation; AV: auditory followed by visual stimulation; N: naïve group (no prior experience with the association of auditory and visual stimuli); E: experienced group (45 minutes of exposure to the association of auditory and visual stimuli, 24 hours prior to experiencing the stimulus sequence). Relative density measures are represented as Mean ± SEM.

A mixed design 2-way ANOVA was performed on the ratio of cell densities of immunopositive neurons to total neurons, with Brain Region as the within-subjects variable and Condition as the between-subjects variable. The Condition variable contained four levels: AV_N_, VA_N_, AV_E_, VA_E_, where N represents naïve, E represents experienced, AV represents auditory followed by visual stimulation, and VA represents visual followed by auditory stimulation. The variable Brain Area contained two levels: AC (auditory cortex) and VC (visual cortex). The ANOVA revealed a significant interaction between Brain Region and Condition (*F*_(3,156,0.05)_ = 38.7; *P* < 0.000001) as well as significant main effects of both Brain Area (*F*_(1,156,0.05)_ = 44.6; *P* < 0.00001) and Condition (*F*_(3,156,0.05)_ = 14.4; *P* < 0.000001). The interaction plot is summarized in Figure 3.

The analysis of Zif268 expression in the visual cortex was restricted to the striate cortex or V1 in order to be able to draw a direct comparison between our findings with those of Zangenehpour and Zatorre [50]. We first compared the modality-specific response of V1 between the two groups of animals (i.e., Experienced vs. Naïve) and found a nonsignificant difference between Zif268 expressions across the two conditions [VA_N_ (mean ± SE = 0.82 ± 0.02) versus VA_E_ (mean ± SE = 0.84 ± 0.02)]. This finding followed our expectation, as we had no *a priori* reason to anticipate a change in V1 activity in response to visual stimuli as a consequence of prior exposure to audiovisual stimuli. The result of this analysis also served as a valuable internal control because it demonstrates that despite individual differences, a great deal of consistency is observed in terms of the extent of cortical activity in response to visual stimuli.

**Figure 4:**
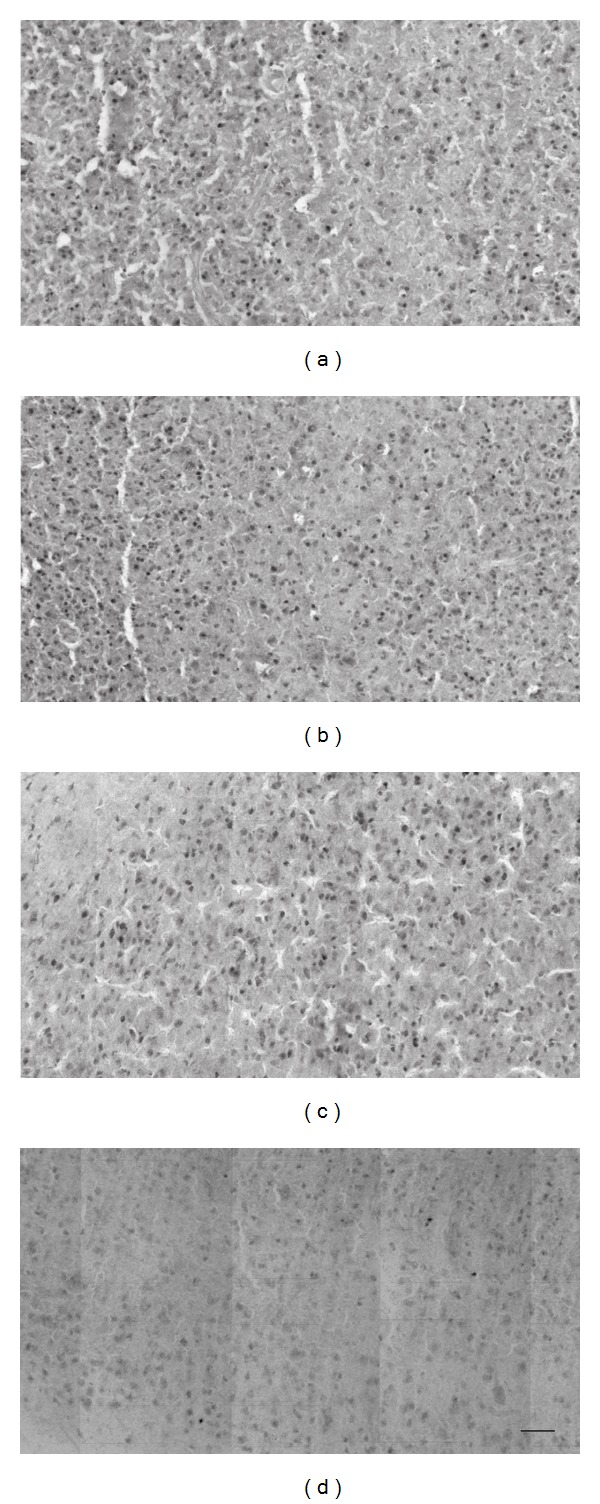
Sample micrographs of Zif268 immunoreactivity in the primary visual cortex (V1). There are a high number of Zif268 protein-positive neurons in V1 in conditions VA_E_ (a), AV_E_ (b), and VA_N_ (c), but not in condition AV_N_ (d). This observation implies that V1 neurons in the animals that were preexposed to audiovisual stimuli were recruited by *both* auditory* and *visual stimuli. The scale bar in (d) represents 50 *μ*m.

We then focused our analyses any cross-modal effects in the visual cortex. We found that V1 expression of Zif268 in condition AV_E_ (mean ± SE = 0.87 ± 0.03) was significantly higher than that found in condition AV_N_ (mean ± SE = 0.57 ± 0.02; *t*_(78,0.05)_ = 8.30; *P* < 0.0001) and nonsignificantly different from that found in conditions VA_N_ and VA_E_. The higher level of Zif268 expression in condition AV_E_ compared to AV_N_ suggests that the auditory component of the stimulation sequence (i.e., auditory followed by visual) was the driving force of V1 protein expression in condition AV_E_, while it had little effect on the activity of V1 in condition AV_N_. These analyses further revealed that the V1 of group E animals responded in the same manner to visual *and* auditory stimuli, while the V1 of group N animals responded in a modality-specific way to the same stimuli. This observation thus constitutes the main finding of our study, namely, that in addition to modality-specific activation of V1 and A1 in all conditions, V1 was crossmodally recruited by auditory stimuli in experienced but not naïve subjects.  Figure 4 shows representative micrographs of the observed activation patterns.

**Figure 5:**
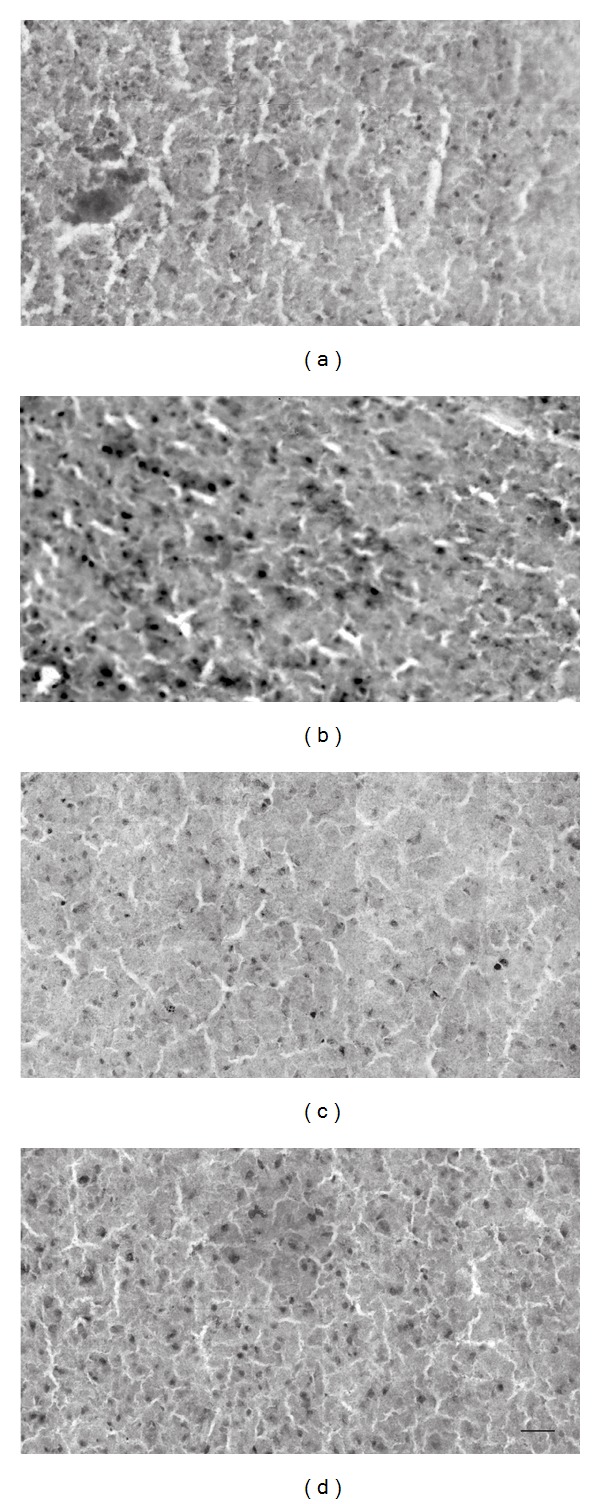
Sample micrographs of Zif268 immunoreactivity in the primary auditory cortex (A1). There are a low number of Zif268 protein-positive neurons in A1 under conditions VA_E_ (a) and VA_N_ (c). Conversely, protein expression appears to be much higher under conditions AV_E_ (b) and AV_N_ (d). Unlike in V1, neurons in A1 appear to be driven in a modality-specific manner (i.e., by auditory stimuli alone) irrespective of the preexposure to audiovisual stimuli. The scale bar in (d) represents 50 *μ*m.

The analysis of Zif268 expression in the auditory cortex was restricted to the core region (also referred to as A1, as defined by [51, 52], e.g.) in order to have an analogous framework for comparisons with V1. Auditory cortical expression of Zif268 in all experimental conditions was found to be modality specific. There was no significant difference between A1 expression of Zif268 in condition AV_N_ (mean ± SE = 0.78 ± 0.04) and that of condition AV_E_ (mean ± SE = 0.78 ± 0.05). Likewise, there was no significant difference between A1 expression of Zif268 in condition VA_N_ (mean ± SE = 0.49 ± 0.02) and that of condition VA_E_ (mean ± SE = 0.47 ± 0.03), suggesting that the auditory component of the stimulation sequence was the only driving force of Zif268 expression in A1.  Figure 5 shows representative micrographs of Zif268 expression obtained from A1 of all four experimental conditions.

Thus far we have been able to find parallel evidence for cross-modal recruitment of visual cortex by auditory stimuli, *only* when those auditory stimuli were experienced *a priori* in the context of a compound audiovisual stimulus, using a nonhuman primate model. As in the Zangenehpour and Zatorre study [50], we did not observe symmetrical cross-modal recruitment; that is, the auditory cortex of the experienced subjects did not show a positive response to visual stimuli. Therefore, having demonstrated this phenomenon at cellular resolution in the nonhuman primate brain, we have provided converging evidence for the findings Zangenehpour and Zatorre in the human brain. The combined findings show that cross-modal recruitment can occur after brief exposure to an audiovisual event in the absence of semantic factors. 

We have also replicated earlier findings in the rat brain [53] regarding the expression profile of Zif268 in the primary visual and auditory cortices following compound stimulation sequences. We confirmed the observations that as a result of auditory followed by visual stimulation, V1 will display baseline protein expression and A1 will display elevated protein expression and that the opposite pattern is obtained if the stimulation sequence is reversed. However, one important difference between our and those earlier findings is the amount of activity-induced protein expression compared to baseline. We found that protein expression in response to stimulation increased by a factor of 1.4 in V1 and by a factor of 1.6 in A1. This increase appears to be lower than the increase found in the rat brain [53], most likely because baseline protein expression in the vervet monkey brain is higher than that in the rat brain. Baseline Zif268 expression can be due to spontaneous translation or stimulus-driven expression caused by spontaneous neural activity [53]. It has previously been found that 30% of neurons in the rat visual cortex are Zif268 immunopositive at baseline [54], whereas we found this to be the case for 57% of neurons in the vervet monkey visual cortex. Although this could be due to interspecies variability, it is also plausible that the mere 3-hour sensory deprivation period in our study design contributed to high levels of baseline protein expression. Rodent studies that report lower baseline expression had sensory deprivation periods of several days to weeks [54, 55]. The sensory deprivation period in primate studies must be limited due to ethical and practical considerations. 

A number of recent functional neuroimaging [32, 33, 35], event-related potential (ERP) recordings [56–60] and magnetoencephalography (MEG [61, 62]) experiments have shown the human auditory cortex to be a site of interaction of audiotactile and audiovisual information. Intracranial recording studies in monkeys [36, 63–67] support those noninvasive human studies by showing that the response of auditory cortical neurons may be influenced by visual and/or somatosensory information. Similarly, both early [68–70] and higher-level visual cortical areas [37, 71–79] have been shown to be under auditory and somatosensory influences. However, there are few studies showing that auditory stimuli alone result in activation of visual cortex (or vice versa) outside of situations involving explicit association or habitual coexposure. In fact, there is considerable evidence for reciprocal inhibition across sensory cortices such that activity in one leads to suppression in the other. The present findings help to clarify the conditions under which cross-modal recruitment may be obtained.

A rich body of literature has been compiled around the topic of cross-modal plasticity in the context of structural manipulations of the nervous system in animal models and sensory deficits in humans. For example, surgical damage of ascending auditory pathways has been shown to lead to the formation of novel retinal projections into the medial geniculate nucleus and the auditory cortex [80–82]. Cross-modal plasticity has been documented in the human brain in the context of sensory deficit, such as the activation of visual cortical areas in blind subjects via tactile [83–87] or auditory tasks [88, 89], or auditory cortex recruitment by visual stimuli in deaf people [90–92]. In the present study, however, we observe a robust cross-modal recruitment of the primary visual cortex (V1) in the absence of similar deprivation-related reorganization. Others have shown the recruitment of extrastriate visual cortical regions after extensive training with visuohaptic object-related tasks [74] and in tactile discrimination of grating orientation [79] in normally sighted individuals. The present study documents that even *primary visual cortex* is subject to cross modal recruitment and that this can happen relatively rapidly. We did not observe symmetric cross-modal recruitment, since we did not detect any activity beyond baseline in A1 in response to the visual stimuli. Given the many studies reviewed above that have shown responsiveness of auditory regions to cross-modal inputs, however, we refrain from interpreting the lack of auditory recruitment as an indication that auditory cortical regions cannot be driven by nonauditory inputs under appropriate conditions.

There is one remaining question: how does V1 receive auditory information such that it is driven by sound? There are three plausible, and not necessarily mutually exclusive, scenarios for such activity to be mediated anatomically (as discussed in [9, 25, 26]): (i) auditory signals may be routed from auditory brainstem nuclei to subcortical visual structures (e.g., lateral geniculate nucleus) and thence to V1; (ii) cortical auditory signals may have influenced V1 indirectly through multimodal cortical regions; (iii) early auditory cortical regions may communicate directly with V1 via corticocortical pathways. There is evidence in support of direct, but sparse, anatomical links between the auditory and striate cortices of the nonhuman primate brain [22, 69, 93]. There is also evidence in support of various thalamic regions, such as the pulvinar [3, 4] and a number of other thalamic nuclei [4], exhibiting multisensory properties. Similar findings linking cortical parts of the brain, such as via the angular gyrus [94], STS [95], and the insular cortex [96], have been used to explain the observed cross-modal plasticity, such as those reported in sighted subjects [97]. Although our molecular mapping approach cannot be used to answer matters pertaining to connectivity directly, it can be combined with traditional tracing approaches in follow-up studies to help find a clearer answer to this question.

## 4. Conclusions

We used molecular activity mapping of the immediate early gene zif268 to further study the nature of cross-modal recruitment of the visual cortex by auditory stimuli following a brief exposure to audiovisual events. When presented with only the auditory or visual components of the bimodal stimuli, naïve animals showed only modality-specific cortical activation. However, animals that had previously been exposed to a combination of auditory *and* visual stimuli showed increased number of active neurons in the primary visual cortex (V1) when presented with sounds alone. As previously implied, this cross-modal activation may be the result of implicit associations of the two stimuli, likely driven by their spatiotemporal characteristics; it was observed after a relatively short period of exposure (~45 min) and lasted for a relatively long period after the initial exposure (~1 day). These new findings suggest that the auditory and visual cortices interact far more extensively than typically assumed. Furthermore, they suggest that the previously reported findings may be directly rooted in the increased activity of the neurons occupying the primary visual cortex.
